# Identification of *CmACL* genes in melon and analysis of their potential functions in fruit sugar and acid accumulation

**DOI:** 10.3389/fpls.2023.1239482

**Published:** 2023-08-15

**Authors:** Kaili Ren, Weiping Kong, Taoxia Tang, Hong Cheng

**Affiliations:** Vegetable Research Institute, Gansu Academy of Agricultural Sciences, Lanzhou, China

**Keywords:** melon, ACL gene family, ATP-citrate lyase, citric acid, fruit

## Abstract

Citric acid is the most important organic acid in melon and has a great influence on fruit flavor quality. ATP-citrate (pro-S) lyase (ACL) is a key regulator in the acetyl-CoA pathway and plays an important role in citric acid metabolism. In this study we analyzed the structure and phylogenetics of *CmACL* genes and their functions in sugar and acid accumulation in melon. A total of four *CmACL* genes were identified in the melon genome, and phylogenetic analysis assigned these genes into the *α* subfamily (*CmACLα1* and *CmACLα2*) and the *β* subfamily (*CmACLβ1* and *CmACLβ2*). Conserved motif and gene structure analyses showed that members of the same subfamily shared identical conserved motifs and gene structures, and probably have similar biological functions. Analysis of *cis*-acting elements revealed that *CmACL* promoter sequences contained regulatory elements related to light, stress, phytohormones, and growth and development, indicating that *CmACL* genes may be involved in melon growth and stress responses. The prediction of protein interaction network showed that CmACL proteins were closely related to the proteins belonging to tricarboxylic acid cycle, glyoxylic acid cycle and glycolytic pathway, suggesting that CmACL proteins may play an important role in sugar and acid metabolism. The expression of *CmACLβ1* was significantly and positively correlated with sucrose content, and *CmACLβ*2 expression was significantly positively correlated with citric acid content, suggesting that *CmACLβ1* and *CmACLβ2* have important roles in sugar and acid accumulation in melon. Our results offer novel insights and avenues for the regulation of sugar and acid levels in melon and provide a theoretical foundation for breeding high-quality melon cultivars.

## 1 Introduction

Melon (*Cucumis melo* L.) is an annual herbaceous trailing plant in the Cucurbitaceae family. Melon fruit is popular and has a fragrant, sweet, and juicy flesh. With the improvement of living standards, high-quality melon is favored by breeders and consumers, and flavor is the most important trait for cultivar improvement and cultivation. Organic acids are a crucial factor controlling the flavor of melon. Moderate levels of organic acids can enhance the flavor of melon, whereas excessive quantities of these acids can adversely affect fruit quality. However, the mechanisms governing organic acid metabolism in melon fruit have not been fully elucidated.

The organic acids in ripe fruits can be dominated by citric acid, malic acid, and tartaric acid (Zhou et al., 2015). The fruits of horticultural plants such as melon (Tang, 2010; Hu, 2018; Tang et al., 2019), citrus (Li et al., 2020a), pineapple (Lu et al., 2013), blueberry (Yu et al., 2016), passion fruit (Zhang et al., 2021), strawberry (Li et al., 2019), and tomato (Wang et al., 2017) mainly contain citric acid. Therefore, the study of citric acid metabolism holds great value for regulating the quality of melon and the fruits of horticultural plants with citric acid as the primary organic acid. Citric acid degradation can occur through three primary pathways: the *γ*-aminobutyric acid pathway, glutamine pathway, and acetyl-CoA pathway. The acetyl-CoA pathway has been extensively investigated in recent years. ATP-citrate (pro-S) lyase (ACL) is a key regulator in citric acid degradation through the acetyl-CoA pathway and converts citric acid into oxaloacetate and acetyl-CoA. The role of ACL in citric acid metabolism has only been documented in a small number of horticultural plants, such as citrus and blueberry (Hu, 2015; Guo, 2020; Li et al., 2020a; Li et al., 2020b). The majority of studies investigating the physiological functions and regulatory mechanisms of ACL have focused on heterologous expression and the model plant *Citrus reticulata*, with no reports on melon. Herein we used bioinformatics analyses to identify *CmACL* genes in melon. We analyzed their phylogenetic relationships, conserved motifs, gene structures, *cis*-acting elements, chromosomal locations, and subcellular locations. The content of soluble sugars and organic acids was measured and correlated to the relative expression of *CmACL* genes. Our results lay a foundation for further investigation into the roles of these genes in sugar and acid accumulation in melon.

## 2 Materials and methods

### 2.1 Data acquisition and identification of CmACL proteins

The AtACL protein sequences of *Arabidopsis thaliana* was retrieved from NCBI (https://www.ncbi.nlm.nih.gov/) and the CitACL protein sequences of *Citrus reticulata* were from the Citrus Pan-genome to Breeding Database of Huazhong Agricultural University (citrus.hzau.edu.cn/orange/) (Hu, 2015; Hu et al., 2015). These sequences were aligned using Blast against the Melon Genomics Database (http://cucurbitgenomics.org/) to search for candidate melon proteins (CmACL proteins). The CmACL proteins were also identified using the conserved domains of ACL proteins. The proteins identified using these two methods were deduplicated and verified by NCBI CDD (http://www.ncbi.nlm.nih.gov/cdd) with an e-value of 10^−4^. The CmACL protein sequences were obtained.

### 2.2 Physicochemical properties of CmACL proteins

The physicochemical properties of the CmACL proteins were determined. Amino acid number, molecular weight, isoelectric point, and total average hydrophilicity were calculated using Expasy (https://www.expasy.org/). The subcellular localization of the CmACL proteins was predicted online using the Cell-PLoc 2.0 package (http://www.csbio.sjtu.edu.cn/bioinf/Cell-PLoc-2/).

### 2.3 Phylogenetic analysis and chromosomal localization of *CmACL* genes

Phylogenetic analysis was performed using the identified CmACL protein sequences and the ACL protein sequences of *A*. *thaliana* and *C*. *reticulata*. Based on multiple sequence alignment, a phylogenetic tree was constructed using the Neighbor-Joining method in MEGA v11.0.13.

The data on the chromosomal locations of the *CmACL* genes were downloaded from the Melon Genomics Database and visualized using an online tool (http://mg2c.iask.in/mg2c_v2.1/).

### 2.4 Conserved motifs and gene structure of *CmACL*

The DNA and CDS sequences of the *CmACL* genes were downloaded from the Melon Genomics Database. The structure of the *CmACL* genes was analyzed using the online tool Gene Structure Display Server 2.0 (http://gsds.gao-lab.org/). The conserved motifs of the CmACL proteins were predicted using MEME 5.5.1 (https://meme-suite.org/meme/tools/meme). The length of the conserved motifs was set to 6–50 amino acids and the number of conserved motifs was set to 20. The 2-kb sequences upstream of the four genes were used as the promoter sequences, and the *cis*-acting elements were identified using PlantCare (http://bioinformatics.psb.ugent.be/webtools/plantcare/html/). Conserved motifs and *cis*-acting elements were visualized with TBtools.

### 2.5 Construction of a CmACL protein interaction network

The interactions of the CmACL proteins were predicted online using STRING (https://cn.string-db.org). The target genes with the highest default Blast scores were selected to construct the protein interaction network. Optimising protein-protein interation networks with Cytoscape (Doncheva et al., 2019; Yu et al., 2022).

### 2.6 Plant materials and growth condition

The melon selfing line Bailangua P7 was provided by Vegetable Research Institute, Gansu Academy of Agricultural Sciences. The plant material were planted in the greenhouse with the day/night temperature of 23–28°C/18–22°C at the melon breeding base in Gaolan county (36°16′49″, 103°37′57″), China, on March 18, 2022. The plant were grown with a 50 cm distance between seedlings and a 60 cm distance between rows and were under the same local management practices (soil management, irrigation, fertilization, and disease control).

Fruit samples were collected at 10, 20, 30, 35, and 40 d after pollination. At each time point, three fruits were harvested from three different plants and used as a replicate, and three replicates were used for the following analyses. The flesh was obtained from the center-equatorial portion of each fruit after removing the pericarp. Then the samples were immediately transferred to liquid nitrogen and stored at –80°C.

### 2.7 Determination of soluble sugars and organic acids in melon

The content of soluble sugars (glucose, fructose, sucrose, raffinose, stachyose, trehalose, maltose, galactose, lactose, trehalose, rhamnose, and arabinose) at different fruit developmental stages was determined using ion chromatography (Thermo iCS5000 ion chromatography system and electrochemical detector).

The content of organic acids (citric acid, malic acid, shikimic acid, oxalic acid, tartaric acid, quinic acid, lactic acid, maleic acid, and succinic acid) at different fruit developmental stages was determined using high-performance liquid chromatography (Waters e2695 separation module and Waters 2998 detector).

### 2.8 Determination of the relative expression of *CmACL* genes in melon

Quantitative real-time (qRT)-PCR was used to determine the relative expression of the *CmACL* genes at different developmental stages in the melon material. Primers were designed using the PrimerQuest Tool with the 18S gene as an internal reference. The primer sequences are shown in **Table 1**. The RNA reverse transcription was performed using the TUREscript 1st Stand cDNA Synthesis Kit (Aidlab), and fluorescence quantification was performed using the abm^®^ EvaGreen qPCR Master Mix-ROX Kit. Each treatment had three technical replicates, and the 2^−ΔΔCt^ method was used to calculate relative gene expression.

**Table 1:** Primer sequences.

Gene name	Primer Sequence (5’- 3’)	
	Forward primer	Reverse primer
*18s*	CAACCATAAACGATGCCGA	AGCCTTGCGACCATACTCC
*CmACLα1*	TATGTTCGGAGAGGTGGT	CCAGTCATTGTGGCTTCT
*CmACLα2*	AGAGCACTCGTGATTGGA	AGCCCTCTCTGGTAGTTG
*CmACLβ1*	CTTGCTGGCAGTGGAATG	GGCGGTATAGAGGCTGTT
*CmACLβ2*	TTGCTGGTAGTGGAATGTTC	CCAAGGATGACGGTAAAGTG

### 2.9 Data analysis

IBM SPSS Statistics 26 software was used for T-test significance analysis and Pearson correlation analysis. Heat map and column chart were generated using GraphPad Prism 9 software.

## 3 Results

### 3.1 Identification and phylogenetic analysis of CmACL proteins in melon

**Figure 1:**
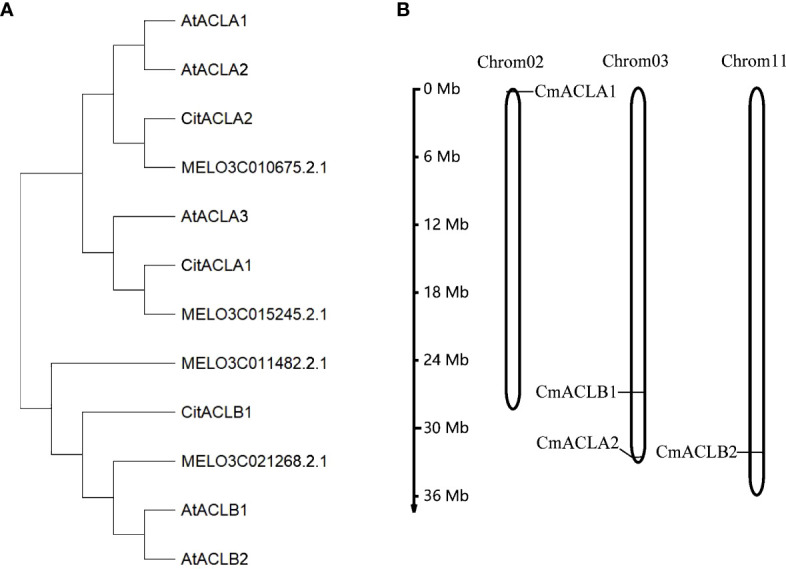
Phylogenetic analysis of ACL **(A)** and chromosomal localization of *CmACL* genes **(B)**.

A total of four CmACL proteins were identified in the melon genome using Blast with the ACL protein sequences of *A*. *thaliana* and *C*. *reticulata* (**Table 1**). To elucidate the phylogenetic relationships of the ACL proteins, a phylogenetic tree was constructed using the Neighbor-Joining method for 12 ACL protein sequences from *A*. *thaliana*, *C*. *reticulata*, and *C*. *melo* (**Figure 1A**). The ACL proteins were divided into *α* and *β* subfamilies. MELO3C015245.2.1 clustered with CitACLα1, and MELO3C010675.2.1 clustered with CitACLα2. Therefore, MELO3C015245.2.1 and MELO3C010675.2.1 were renamed as *CmACLα1* and *CmACLα2*, respectively. MELO3C011482.2.1 and MELO3C021268.2.1 clustered with the ACL proteins in the *β* subfamily and were renamed as *CmACLβ1* and *CmACLβ2*.

Chromosomal localization (**Figure 1B**) showed one *CmACL* gene on Chr2, one on Chr11, and two on Chr3. The number of amino acids was 423 and 608 in the *α* and *β* subfamilies, respectively (**Table 2**). The relative molecular weight was 46653.68–66017.74 Da, and the isoelectric point was 5.34–8.23. The hydrophilicity ranged between −0.124 and −0.036, and the four CmACL proteins were hydrophilic. The instability index ranged between 30.52 and 42.15, indicating that the CmACL proteins were stable except for CmACLα1. The four CmACL proteins were localized in the nucleus.

**Table 2:** Physical and chemical properties of CmACL.

Name	Gene ID	Chromosome distribution	Amino acids(aa)	Molecular weight(Da)	pI	GRAVY	Instability index	hmm name	Subcellular localization
*CmACLα1*	MELO3C015245.2.1	chr02: 229253. 235408 (-)	423	46780.86	5.44	-0.087	42.15	PLN02235	Mitochondrion
*CmACLα2*	MELO3C010675.2.1	chr03: 31551192. 31555805 (-)	423	46653.68	5.34	-0.124	39.54	PLN02235	Mitochondrion
*CmACLβ1*	MELO3C011482.2.1	chr03: 25714081. 25722118 (-)	608	65957.78	8.23	-0.036	30.52	PLN02522	Mitochondrion
*CmACLβ2*	MELO3C021268.2.1	chr11: 30776928. 30782529 (+)	608	66017.74	7.59	-0.044	32.73	PLN02522	Mitochondrion

### 3.2 Conserved motifs and gene structure of *CmACL*

**Figure 2:**
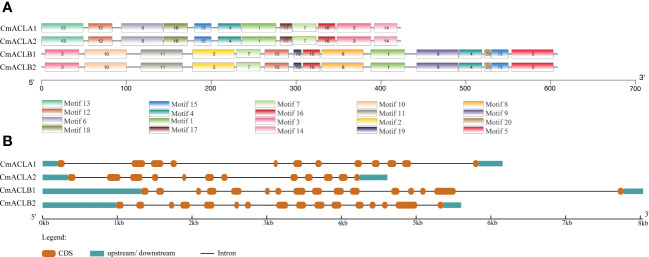
Conserved motifs of CmACL proteins **(A)** and structures of *CmACL* genes **(B)**.

The function of a protein is determined by its number and type of conserved motifs. The conserved motifs of the CmACL proteins were predicted by MEME and visualized using TBtools. As shown in **Figure 2A**, both CmACLα1 and CmACLα2 of the *α* subfamily contained 12 conserved motifs with similar positions, including motif1, motif3, motif4, motif6, motif7, motif12, motif13, motif14, motif15, motif16, motif17, and motif18. Both CmACLβ1 and CmACLβ2 of the *β* subfamily contained 15 conserved motifs with similar positions, including motif1, motif2, motif3, motif4, motif5, motif7, motif8, motif9, motif10, motif11, motif12, motif15, motif16, motif19, and motif20. Analysis of the conserved motifs in the CmACL proteins showed that the members in the same subfamily shared identical conserved motifs indicating that the same subfamily might have similar biological functions.

The structures of four *CmACL* genes were analyzed to further reveal their phylogenetic diversity (**Figure 2B**). The results showed that the genes in the same subfamily shared similar structures. *CmACLα1* and *CmACLα2* contained 11 introns and 12 exons, while *CmACLβ1* and *CmACLβ2* contained 15 introns and 16 exons. The variances in intron and exon distribution may be attributed to evolution, as distinct gene structures have led to different gene functions.

### 3.3 Analysis of *CmACL cis*-acting elements and protein interaction network

**Figure 3:**
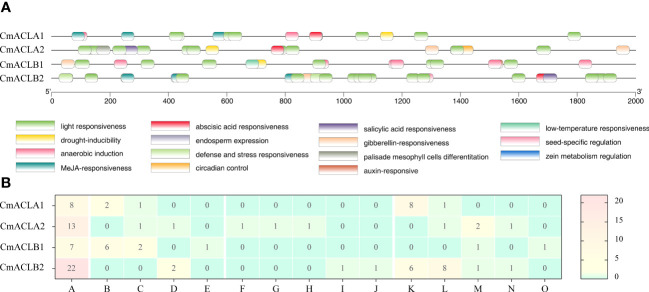
Distribution **(A)** and number **(B)** of *cis*-acting elements in *CmACL* genes.

The *cis*-acting elements of the *CmACL* genes are shown in **Figure 3**. These elements were related to light, abiotic and biotic stress, plant growth and development, and phytohormone. Light responsiveness elements were most abundant and present in the four *CmACL* genes. Plant abiotic and biotic stress elements included anaerobic induction (*CmACLα1* and *CmACLβ1*), drought-inducibility (*CmACLα1/2 and CmACLβ1*), defense and stress responsiveness (*CmACLα2* and *CmACLβ2*), and low-temperature responsiveness (*CmACLβ1*). Plant growth and development elements included circadian control (*CmACLα2*), palisade mesophyll cells differentiation (*CmACLα2*), endosperm expression (*CmACLα2*), seed-specific regulation (*CmACLβ2*), and zein metabolism regulation (*CmACLβ2*). Phytohormone elements included MeJA-responsiveness (*CmACLα1* and *CmACLβ2*), abscisic acid responsiveness (*CmACLα1*/*2* and *CmACLβ2*), gibberellin-responsiveness (*CmACLα2* and *CmACLβ1*/*2*), salicylic acid responsiveness (*CmACLα2* and *CmACLβ2*), and auxin-responsive (*CmACLβ1*). These results suggest that *CmACL* genes may have important roles in plant growth, development, and the stress response.

**Figure 4:**
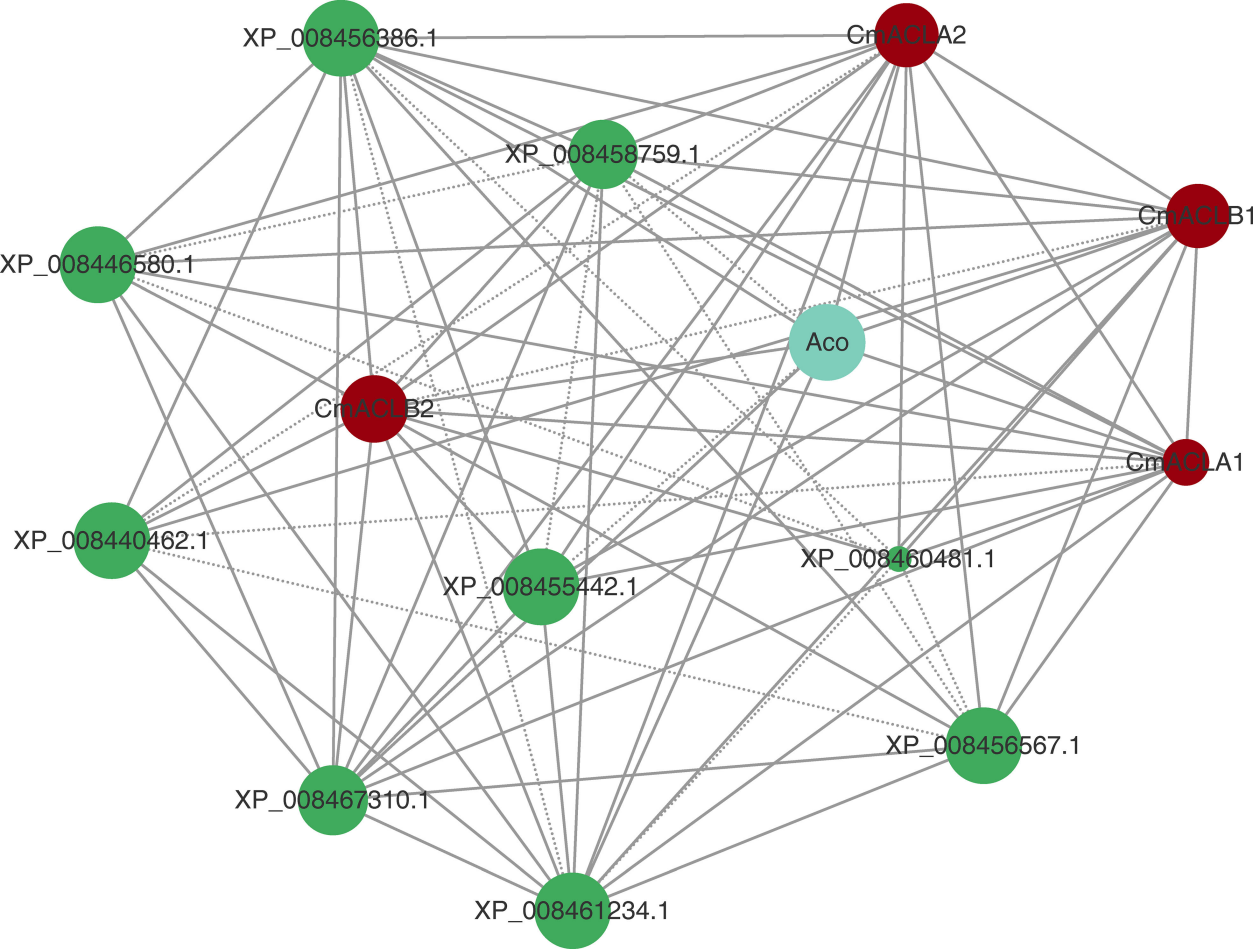
Protein-protein interaction network. The area of the circles represents the number of interacting proteins, the larger area of the circles indicate more interacting proteins. The dashed lines represent protein interaction score range from 0.7 to 0.9, and solid lines means protein interaction score>0.9.

The protein interaction network was constructed using the online tool STRING (**Figure 4**). Results showed that all four CmACL proteins interacted with one another. Notably, several proteins such as Aco, XP_008467310.1, XP_008455442.1, XP_008456386.1, XP_008461234.1, XP_008456567.1, XP_008440462.1, XP_008460481.1 and XP_008446580.1, belonging to tricarboxylic acid cycle, glyoxylate cycle, and glycolytic pathway, were showing tight relationships with CmACL, suggesting that CmACL may play an important role in sugar and acid metabolism.

### 3.4 Soluble sugar and organic acid content in melon fruit

The levels of soluble sugars and organic acids in Bailangua P7 were determined at 10, 20, 30, 35, and 40 d after pollination. **Table 3** shows that the melon contained high levels of sucrose, fructose, and glucose; low levels of raffinose, stachyose, trehalose, and maltose; and no detectable levels of galactose, lactose, trehalose, rhamnose, and arabinose. Fructose and glucose increased as the fruit ripened, maximized at 35 d after pollination, and then decreased slightly at 40 d post-pollination. Sucrose was low from 10 to 30 d and accumulated rapidly from 35 to 40 d after pollination, peaking at 40 d post-pollination. **Table 4** shows that the melon fruit contained high levels of citric acid and malic acid; low levels of shikimic acid; and no detectable levels of oxalic acid, tartaric acid, quinic acid, lactic acid, maleic acid, and succinic acid. Citric acid was the highest at 10 d after pollination, decreased rapidly from 10 to 20 d, and then increased and decreased from 20 to 40 d. Malic acid decreased with fruit ripening.

**Table 3:** Soluble sugars at different fruit developmental stages.

Soluble sugar	B10	B20	B30	B35	B40
Glucose(μg/mg)	13.54 ± 0.33e	14.91 ± 0.31d	19.76 ± 0.53c	23.69 ± 0.70a	20.97 ± 0.59b
Fructose(μg/mg)	21.84 ± 0.65d	23.54 ± 0.64d	32.55 ± 1.01c	40.76 ± 1.39a	37.17 ± 1.56b
Sucrose(μg/mg)	3.02 ± 0.07c	0.46 ± 0.02d	2.47 ± 0.07c	7.38 ± 0.25b	42.06 ± 1.54a
Raffinose(μg/mg)	0.19 ± 0.01a	0.06 ± 0.00c	0.08 ± 0.00b	0.06 ± 0.00c	0.06 ± 0.00c
Stachyose(μg/mg)	2.06 ± 0.06a	0.28 ± 0.01c	0.33 ± 0.01b	0.38 ± 0.02b	0.21 ± 0.01d
Trehalose(μg/mg)	0.21 ± 0.00c	0.06 ± 0.00e	0.16 ± 0.01d	0.26 ± 0.01b	0.41 ± 0.01a
Maltose(μg/mg)	0.00 ± 0.00c	0.00 ± 0.00c	0.00 ± 0.00c	0.08 ± 0.00b	0.18 ± 0.01a
Galactose(μg/mg)	–	–	–	–	–
Lactose(μg/mg)	–	–	–	–	–
Trehalose(μg/mg)	–	–	–	–	–
Rhamnose(μg/mg)	–	–	–	–	–
Arabinose(μg/mg)	–	–	–	–	–

B10–B40 represent the sampling days at 10–40 d after pollination; The detect data are presented as mean ± standard error and “−” indicates levels below the detection limit; The difference lowercase letters indicate statistically significant differences at P ≤ 0.05.

**Table 4:** Organic acids at different fruit developmental stages.

Organic acids	B10	B20	B30	B35	B40
Citric acid(μg/g)	18788.79 ± 509.18a	1779.84 ± 49.74d	3037.38 ± 24.14b	3272.47 ± 66.05b	2290.13 ± 73.16c
Malic acid(μg/g)	2851.70 ± 173.47a	2200.28 ± 39.82b	556.01 ± 4.97c	377.57 ± 3.16d	374.22 ± 7.27d
Shikimic acid(μg/g)	66.02 ± 0.60a	1.13 ± 0.04d	3.49 ± 0.01c	3.95 ± 0.10c	4.68 ± 0.22b
Oxalic acid(μg/g)	–	–	–	–	–
Tartaric acid(μg/g)	–	–	–	–	–
Quinic acid(μg/g)	–	–	–	–	–
Lactic acid(μg/g)	–	–	–	–	–
Maleic acid(μg/g)	–	–	–	–	–
Succinic acid(μg/g)	–	–	–	–	–

B10–B40 represent the sampling days at 10–40 d after pollination; The detect data are presented as mean ± standard error and “−” indicates levels below the detection limit; The difference lowercase letters indicate statistically significant differences at P ≤ 0.05.

### 3.5 Relative expression of *CmACL* genes in melon fruit

**Figure 5:**
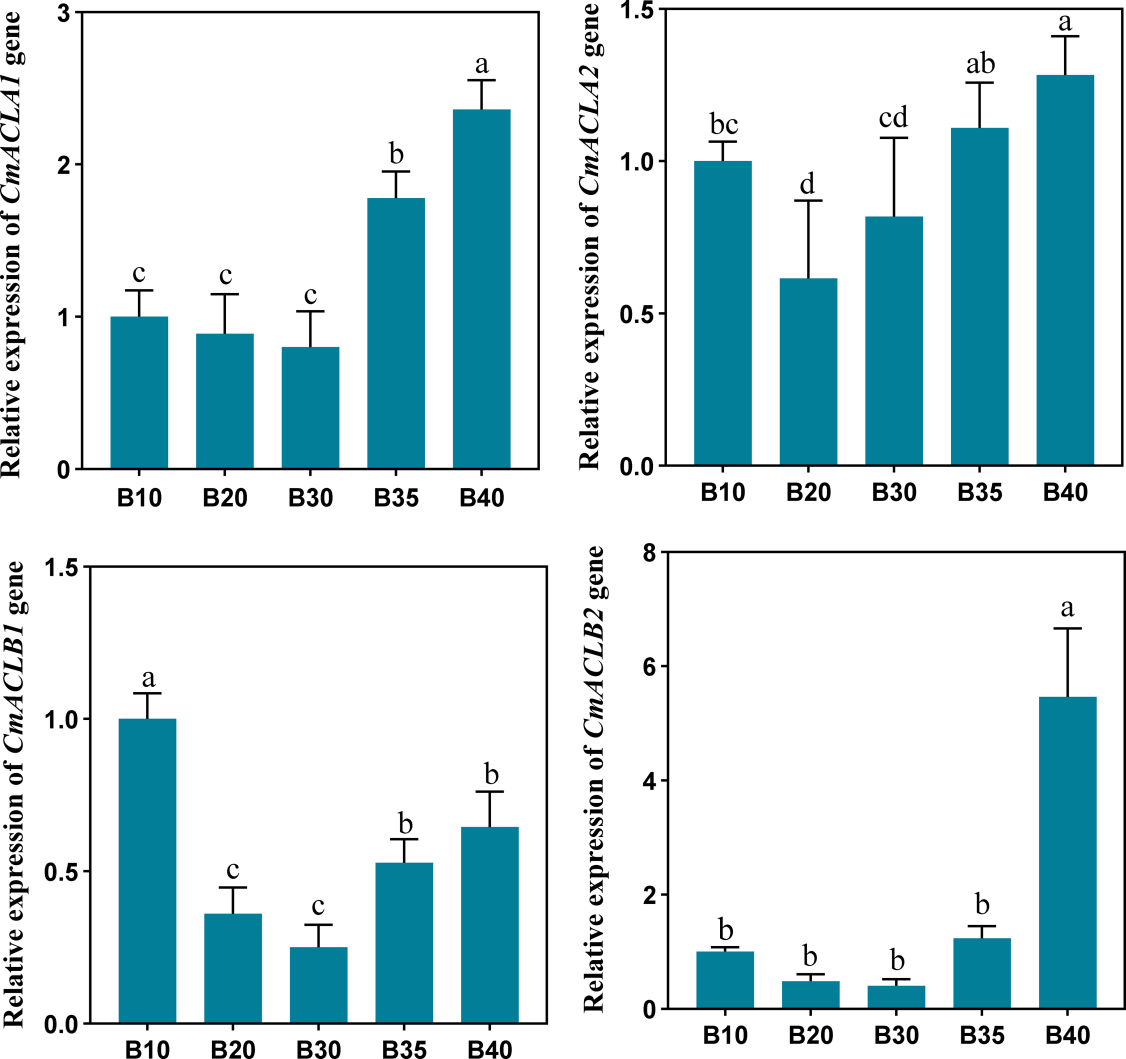
Relative expression of *CmACL* genes. The difference lowercase letters indicate statistically significant differences at P ≤ 0.05.

The relative expression of *CmACL* genes was determined at different fruit developmental stages (**Figure 5**). The expression of four *CmACL* genes decreased and then increased with fruit ripening. The expression of *CmACLα2* decreased from 10 to 20 d and then gradually increased from 20 to 40 d. The expression of *CmACLα1*, *CmACLβ1*, and *CmACLβ2* decreased from 10 to 30 d and then increased from 30 to 40 d. The expression of *CmACLα1* at 40 d after pollination was 2.36-, 2.65-, 2.95-, and 1.33-fold that at 10, 20, 30, and 35 d. The expression of *CmACLβ1* at 40 d after pollination was 5.46-, 11.38-, 13.65-, and 4.40-fold that at 10, 20, 30, and 35 d.

### 3.6 Correlation of *CmACL* gene expression with sugar and acid content

The expression of *CmACL* genes was correlated with the content of sugars and acids in melon (**Table 5**). The results showed significant correlations between the expression of four genes. For example, the expression of *CmACLα1* was significantly and positively correlated with *CmACLα2* and *CmACLβ1* (correlation coefficients of 0.824 and 0.841, respectively), and the expression of *CmACLα2* was significantly and positively correlated with *CmACLβ1* (0.665).

**Table 5:** Correlation of *CmACL* gene expression with sugar and acid levels in fruit.

	*ACLα1*	*ACLα2*	*ACLβ1*	*ACLβ2*
*ACLα1*	1	0.824**	0.841**	0.232
*ACLα2*	0.824**	1	0.665**	0.459
*ACLβ1*	0.841**	0.665**	1	0.235
*ACLβ2*	0.232	0.459	0.235	1
Citric acid	-0.289	0.077	-0.200	0.822**
Malic acid	-0.584*	-0.430	-0.418	0.482
Shikimic acid	-0.259	0.089	-0.155	0.843**
Glucose	0.614*	0.477	0.349	-0.353
Fructose	0.687**	0.545*	0.440	-0.278
Sucrose	0.867**	0.695**	0.970**	0.200
Raffinose	-0.354	0.018	-0.211	0.765**
Stachyose	-0.323	0.036	-0.241	0.806**
Trehalose	0.884**	0.859**	0.866**	0.395
Maltose	0.961**	0.748**	0.907**	0.151

*indicates a significant correlation between the two indicators (P ≤ 0.05); **indicates a highly significant correlation between the two indicators (P ≤ 0.01).

There were significant correlations between the expression of *CmACL* genes and sugar and acid content. The expression of *CmACLα1* was significantly negatively correlated with malic acid (−0.584) and significantly positively correlated with glucose, fructose, sucrose, trehalose, and maltose (0.614, 0.687, 0.867, 0.884, and 0.961). The expression of *CmACLα2* was significantly positively correlated with fructose, sucrose, trehalose, and maltose (0.545, 0.695, 0.859, and 0.748). The expression of *CmACLβ1* was significantly positively correlated with sucrose, trehalose, and maltose (0.970, 0.866, and 0.907). The expression of *CmACLβ2* was significantly positively correlated with citric acid, shikimic acid, raffinose, and stachyose (0.822, 0.843, 0.765, and 0.806).

## 4 Discussion

The ACL proteins can be divided into *α* and *β* subfamilies based on conserved domains. In *Lupinus albus* and *Saccharum officinarum*, two genes were identified that encode proteins in the ACLα and ACLβ subfamilies, respectively (Langlade et al., 2002; Li et al., 2012). In *A*. *thaliana*, three genes encode ACLα proteins and two genes encode ACLβ proteins (Fatland et al., 2002). In *C*. *reticulata*, two genes encode ACLα proteins and one gene encodes an ACLβ protein (Wang, 2018). In the present study, four *CmACL* genes, including two *α* subfamily genes (*CmACLα1* and *CmACLα2*) and two *β* subfamily genes (*CmACLβ1* and *CmACLβ2*), were identified in melon by whole-genome analysis using the ACL protein sequences of *A*. *thaliana* and *C*. *reticulata*. Physicochemical characterization suggested that proteins in the same subfamily had identical numbers of amino acids (423 in the *α* subfamily and 608 in the *β* subfamily) and similar molecular weights (46653.68–46780.86 Da in the *α* subfamily and 65957.78–66017.74 Da in the *β* subfamily). These findings indicate different structures of proteins in different subfamilies. Subcellular localization showed that the four CmACL proteins were localized in the mitochondria, which differs from previous studies whereby ACL proteins were found to be localized in the plastid, cytoplasm, and nucleus (Rangasamy and Ratledge, 2000; Guo, 2020), which might be explained by the different species used in these studies. Analyses of gene structure and protein conserved motifs showed identical gene structures and conserved motifs in the same subfamily, indicating that they may have similar biological functions.

Plant ACL proteins are involved in many biological processes and have different functions in plants. These ACL proteins have important roles in plant growth and development. For instance, ACL overexpression promoted the accumulation of wax in *A*. *thaliana* stems, rubber and triterpenes in *Taraxacum brevicorniculatum* roots, and anthocyanins in *Paeonia lactiflora* petals, by increasing the abundance of their precursor acetyl-CoA (Go et al., 2014; Xing et al., 2014; Luan et al., 2022). The ACL proteins also play important regulatory roles in plant responses to stresses such as drought. The *SoACL* genes in *S*. *officinarum* and *CitACL* genes in *C*. *reticulata* were reported to respond to drought and abscisic acid (Crifò et al., 2012; Li et al., 2012; Lo Piero et al., 2014; Hu et al., 2015; Phan et al., 2017). Promoter sequences usually contain *cis*-acting elements with specific functions that reflect the potential functions of genes (Zhang et al., 2019). Analysis of the *CmACL* gene promoters identified elements related to light, stress response, plant growth and development, and phytohormone response. Moreover, the functions of ACL proteins in plant growth, development, and stress response have been widely documented in other plants. Therefore, we speculate that *CmACL* genes may have important roles in regulating melon growth, development, and the stress response. Furthermore, the prediction of protein interaction network showed that CmACL proteins were closely related to the proteins belonging to tricarboxylic acid cycle, glyoxylic acid cycle and glycolytic pathway, suggesting that CmACL protein may play an important role in sugar and acid metabolism.

The sugars and acids in melon determine fruit flavor quality. We measured the soluble sugar and organic acid contents in melon fruit. The results showed that the fruit mainly contained sucrose and citric acid, which is consistent with previous studies (Hu, 2018; Zhang et al., 2022). We also measured the relative expression of *CmACL* genes and found that their expression decreased and then increased with fruit ripening. The expression of *CmACLα1*, *CmACLα2*, and *CmACLβ1* was significantly and positively correlated with sucrose and the highest correlation was *CmACLβ1*, with a correlation coefficient of 0.970. The expression of *CmACLβ2* was significantly and positively correlated with citric acid, and its correlation coefficient was 0.843. Therefore, *CmACLβ1* and *CmACLβ2* may serve important roles in sugar and acid accumulation in melon fruit. *CitACLα1* in *C*. *reticulata* (Li et al., 2020a) and *VcACL* in *Vaccinium corymbosum* (Li et al., 2020b) were reported to be involved in citric acid metabolism. The role of ACL in fruit sucrose regulation has not been reported. The present study provides a reference and new approach for further investigations of *CmACL* genes in sugar and acid metabolism in melon, providing a theoretical foundation for breeding high-quality melon cultivars.

## 5 Conclusion

In this study, four *CmACL* genes were identified in melon genome and divided into two subfamily according to phylogenetic relationships, and members of the same subfamily had similar gene structure and conserved domain. The promoter sequences of *CmACL* genes contained regulatory elements related to light, stress, phytohormones, and growth and development, and the protein interaction network prediction showed that CmACL proteins were closely related to the proteins belonging to tricarboxylic acid cycle, glyoxylic acid cycle and glycolytic pathway. Correlation analysis was conducted between *CmACL* genes expression and sugar/acid content. Taken together, the *CmACL* genes that potentially regulated sugar and acid metabolism were mined. The results expand the understanding of *CmACL* genes in melon and provide a reference for further investigation of their functions in sugar and acid accumulation.

## Data availability statement

The datasets presented in this study can be found in online repositories. The names of the repository/repositories and accession number(s) can be found in the article/**Supplementary Material**.

## Author contributions

KR and HC designed the experiments. KR, WK and TT performed the experiments. KR analyzed the data, and wrote the manuscript. HC helped revise the manuscript. All authors contributed to the article and approved the submitted version.
