# Supplementary material for: Identification of CmACL genes in melon and analysis of their potential functions in fruit sugar and acid accumulation
Source: Front Plant Sci. 2023 Aug 15;14:1239482. doi: 10.3389/fpls.2023.1239482 (PMC10465347; doi:10.3389/fpls.2023.1239482)
Supplement: Supplementary file 1 [file Table_1.docx]

The AtACL protein sequences of *Arabidopsis thaliana* were retrieved from NCBI (<https://www>.ncbi.nlm.nih.gov/).

**AtACLA-1**

gene ID: 837610

accession number: NP_172537.1/NP_001184954.1/NP_849634.1

**AtACLA-2**

gene ID: 842375

accession number: NP_176280.1/NP_001320572.1

**AtACLA-3**

gene ID: 837466

accession number: NP_172414.1

**AtACLB-1**

gene ID: 819845

accession number: NP_187317.1/NP_001326324.1

**AtACLB-2**

gene ID:835006

accession number: NP_001332247.1/NP_199757.1

The CitACL protein sequences of *Citrus reticulata* were from the Citrus Pan-genome to Breeding Database of Huazhong Agricultural University (citrus.hzau.edu.cn/orange/)

**CitACLα1**

Gene ID: Cs6g01210

transcript ID: Cs6g01210.2

**CitACLα2**

Gene ID: Cs7g08950

transcript ID: Cs7g08950.2

**CitACLβ1**

Gene ID: Cs9g02230

transcript ID: Cs9g02230.2
